# Change processes in cognitive therapy for social anxiety disorder: A comparison of face-to-face and internet-based treatment formats

**DOI:** 10.1016/j.invent.2024.100786

**Published:** 2024-11-02

**Authors:** Graham R. Thew, Anke Ehlers, David M. Clark

**Affiliations:** a Department of Experimental Psychology, University of Oxford, The Old Rectory, Paradise Square, Oxford OX1 1TW, UK; b Oxford University Hospitals NHS Foundation Trust, Oxford, UK; c Oxford Health NHS Foundation Trust, Oxford, UK

**Keywords:** Social anxiety, Cognitive therapy, Multilevel model, Structural equation model, change processes, mediation

## Abstract

**Background:**

Compared to efficacy research, studies investigating the processes of change in psychological therapy are rare, especially for internet-based interventions. While many online therapies are based on face-to-face therapy protocols, it is unknown whether the processes of clinical improvement differ between these treatment formats.

**Objective:**

To examine candidate change processes in an online therapist-guided cognitive therapy intervention for social anxiety disorder (iCT-SAD), and compare the results to the corresponding face-to-face therapy (CT-SAD).

**Methods:**

Data from a randomised controlled trial (*n* = 99) were analysed using Multilevel Structural Equation Models, incorporating the temporal precedence of the process variable, and disaggregating the within- and between-subject components of the predictors. These examined three candidate change processes: self-focused attention, negative social cognitions, and depressed mood. Moderated mediation models provided an additional test of the moderating effect of treatment format.

**Results:**

Negative social cognitions and self-focused attention were supported as significant mediators of clinical improvement in both CT-SAD and iCT-SAD. Effects were of similar strength and moderated mediation was not observed. There was also evidence of cyclical relationships between social anxiety symptoms and these process variables. Depressed mood also emerged as a significant but weak mediator in CT-SAD, but not in iCT-SAD. Moderated mediation was not observed.

**Conclusion:**

The online format of therapy showed a similar pattern of change processes to face-to-face treatment, with self-focused attention and negative social cognitions mediating clinical improvement in both treatments. Efforts to improve the efficacy and efficiency of SAD interventions by targeting these factors may therefore be equally applicable to online and face-to-face interventions.

## Highlights

• We compared change processes across face-to-face and internet-based therapy formats.
• Negative cognitions and self-focused attention mediated outcomes in both formats.
• Depressed mood showed weak mediation in CT-SAD and no mediation in iCT-SAD.
• The online treatment showed similar patterns of change to face-to-face therapy.

## 1 Introduction

Studies of the efficacy of internet-based therapies over recent decades have generally shown good evidence of their efficacy in the treatment of mental health problems, particularly anxiety disorders (Andersson et al., 2018, Andersson et al., 2019; Andrews et al., 2010; Bennett et al., 2020; Hedman et al., 2012; Peñate and Fumero, 2015). However, the reasons why these interventions work and the mechanisms through which they operate are less clearly understood. Internet interventions are not unique in this respect, and many researchers have highlighted a significant need for more research on mediators and moderators of clinical improvement in both face-to-face and online therapies (Andersson et al., 2009; Holmes et al., 2018; Kazdin, 2007).

Given than many internet-based psychological therapies are derived from face-to-face therapy protocols, it might be assumed that because the content is similar between the two formats, the change processes underlying clinical improvement will also be the same. However, no studies to date have compared change processes between an internet therapy and its equivalent face-to-face counterpart. Hedman et al. (2013) compared mediators across two formats of face-to-face cognitive therapy (CT) for social anxiety disorder (SAD): individual and group. Different patterns of mediation were shown across the two, suggesting that the individual and group therapies may have been having their clinical effects through different mechanisms. In this study, a pure comparison of treatment formats was not possible given the two interventions were based on different theoretical models, but the findings emphasise that mediators may differ across therapy formats and it is therefore important to investigate this empirically. Finding that the change processes in online and face-to-face therapies are similar would suggest it is the content, rather than format, that is most important for driving clinical improvement. It would also mean that efforts to target these clinically to improve treatment efficacy and efficiency may be equally applicable across formats. If the change processes differ, this might suggest that online programmes may not be having their clinical effects in the same way, and that efforts to improve them may need to diverge from their face-to-face counterparts.

Internet-based Cognitive Therapy for Social Anxiety Disorder (iCT-SAD) is a therapist-guided online intervention developed in the UK (see Stott et al., 2013). It implements the content and procedures of the Clark and Wells SAD face-to-face therapy protocol (CT-SAD) and is based on this theoretical model (Clark and Wells, 1995). The model suggests that negative social cognitions and self-focused attention are key processes that maintain SAD, meaning they may act as mediators of changes in social anxiety symptoms. Although the number of studies investigating mediation in face-to-face SAD interventions is limited, there is some evidence to support cognitions (Boden et al., 2012; Calamaras et al., 2015; Goldin et al., 2014; Gregory et al., 2018; Hoffart et al., 2012; Thew et al., 2020), and self-focused attention (Hedman et al., 2013; Mörtberg et al., 2015; Thew et al., 2020) as mediators of clinical improvement.

The aim of the present study was therefore to investigate change processes during iCT-SAD, examining negative social cognitions and self-focused attention given their strong theoretical rationale and existing evidence from face-to-face studies. Depressed mood, which is not part of the Clark and Wells (1995) model, but has been examined in other studies (Moscovitch et al., 2005; Shalom et al., 2024), was also examined as a control variable. By comparing the findings with a second group of participants who had undertaken the equivalent treatment face-to-face (CT-SAD), the study aimed to examine whether the format of therapy was associated with differential effects of candidate treatment processes.

## 2 Method

### 2.1 Design

This study analysed data from a randomised controlled trial of Cognitive Therapy for SAD (Clark et al., 2023). The trial compared three conditions: face-to-face Cognitive Therapy (CT-SAD), internet-based Cognitive Therapy (iCT-SAD), and a waitlist control group. Participants in the waitlist condition were subsequently randomised to one of the two active treatments. The results of this trial indicated both CT-SAD and iCT-SAD were superior to waitlist, with no significant differences found between the two regarding social anxiety outcomes. Midtreatment scores on a composite measure of SAD process variables (including negative social cognitions, safety behaviours, social attitudes and self-focused attention) were found to mediate posttreatment social anxiety scores. The present study used week-to-week data to explore this in greater depth.

### 2.2 Participants

Data from all 99 participants from the randomised controlled trial were analysed. All had received either face-to-face CT-SAD (*n* = 50) or internet-based iCT-SAD (*n* = 49). Participants were recruited from local clinical services and self-referrals from the community. All provided informed consent to participate. Inclusion criteria were: meeting DSM-IV (American Psychiatric Association, 2000) criteria for SAD; SAD is the main problem; age 18–65 years; internet access from home; no psychotropic medication or a stable dose for at least two months without improvement and willing to keep the dose constant. Exclusion criteria were: unable to attend weekly sessions; previous CBT or exposure therapy for SAD; immediate suicide risk; current substance dependency; current or past psychosis, borderline personality disorder. The study was reviewed and approved by the local ethics committee, and all procedures complied with relevant laws and institutional guidelines.

### 2.3 Treatments

The face-to-face CT-SAD protocol was based on the Clark and Wells (1995) model, and is the first-line recommended treatment for SAD according to NICE guidelines (NICE, 2013). Treatment materials, resources and training videos are available at www.oxcadatresources.com. The iCT-SAD intervention implements the full content of CT-SAD, presented online in a series of treatment modules that include psychoeducation, written and behavioural tasks, videos demonstrating key behavioural experiments or treatment principles, case examples, and patient testimony. Each participant is assigned a therapist, with whom they communicate via asynchronous messaging from within the programme, weekly phonecalls, and webcam chats. The website also contains a library of therapy resources, an online log where people can plan and record the results of behavioural experiments, and videos of virtual audiences where people can record themselves giving presentations. Further details of the programme are given in Stott et al. (2013) and Clark et al. (2023). Both treatments lasted 14 weeks, followed by three booster sessions at monthly intervals.

### 2.4 Therapists

Both treatments were delivered by three highly experienced cognitive therapists, with each therapist delivering both CT-SAD and iCT-SAD interventions, receiving clinical supervision throughout.

### 2.5 Measures

Measures were completed on a weekly basis throughout the treatment, prior to each session in the CT-SAD group, and online in the iCT-SAD group.

#### 2.5.1 Liebowitz Social Anxiety Scale – Self Report version (LSAS-SR; Baker et al., 2002)

A 24-item scale describing a range of social situations. Respondents give two scores per item in relation to the last week; firstly, the extent of fear or anxiety they would experience in the situation, and secondly, how much they would tend to avoid the situation. The scale has good psychometric properties for both clinician-rated and self-report versions (Fresco et al., 2001; Heimberg et al., 1999). Cronbach's alpha in the present sample was 0.90 at baseline and 0.96 at posttreatment.

#### 2.5.2 Social Cognitions Questionnaire (SCQ; Clark, 2005, available at www.oxcadatresources.com)

This scale presents 22 negative social cognitions, each of which is rated for both the frequency with which it occurred in the last week when the respondent was anxious (rated from 1 = thought never occurs to 5 = thought always occurs when I am nervous), and the degree to which they believe the thought to be true when it occurs (rated from 0 = I do not believe this thought, to 100 = I am completely convinced this thought is true). Scores for the Frequency and Belief subscales are calculated separately. Cronbach's alpha in the present sample was 0.88 at baseline and 0.91 at posttreatment for the frequency subscale and 0.86 at baseline and 0.94 at posttreatment for the belief subscale.

#### 2.5.3 Self-focused attention

This was measured using the mean of the two self-focused attention items on the Social Phobia Weekly Summary Scale (SPWSS; Clark et al., 2003, available at www.oxcadatresources.com). The SPWSS is a six-item scale exploring the respondent's social anxiety over the previous week. In addition to items on self-focused attention (in general, and in more difficult social situations), the scale elicits ratings of avoidance, anticipatory worry, post-event rumination, and overall perceived social anxiety. All items are rated on 0–8 Likert scales. Cronbach's alpha in the present sample was 0.74 at baseline and 0.94 at posttreatment.

#### 2.5.4 Patient health questionnaire – 9-item version (PHQ; Kroenke et al., 2001)

A widely used and validated assessment of depression symptom severity over the previous two weeks; scores ≥5 are clinically significant, with scores 5–9, 10–14, 15–19, and 20 and above reflecting mild, moderate, moderately severe, and severe depression, respectively (Maximum score 27). Cronbach's alpha in the present sample was 0.83 at baseline and 0.84 at posttreatment.

### 2.6 Analysis

Mediation was examined using multilevel structural equation models (see Preacher et al., 2010, Preacher et al., 2011) following the approach of Mörtberg et al. (2015) and Thew et al. (2020), with scores at each session (level 1) nested within participants (level 2). The number of days in treatment was the independent variable, and LSAS score (social anxiety) was the dependent variable. Replicating the analyses of Thew et al. (2020), three candidate process variables were assessed: 1) self-focused attention, measured using the mean of the two self-focused attention items of the SPWSS; 2) negative social cognitions, measured using the SCQ. A composite z score was computed from the two SCQ subscales to incorporate both the frequency of, and belief in, these cognitions, and to reduce the number of models required; 3) depressed mood, measured using the total PHQ score.

**Fig. 1:**
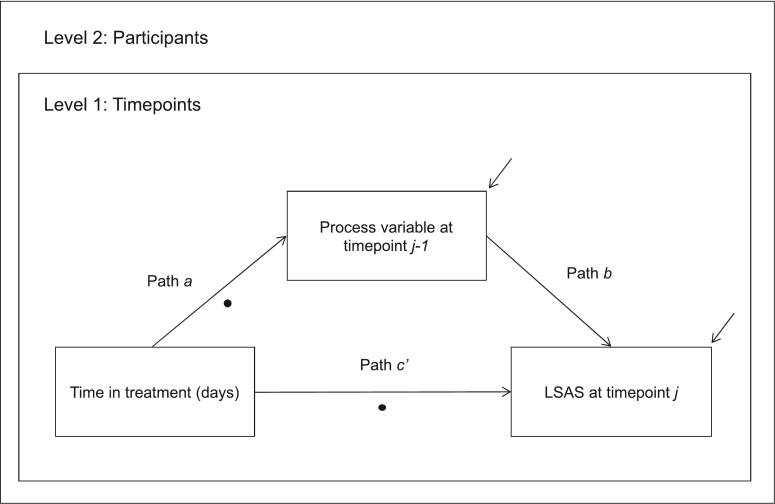
Simplified path diagram of multilevel structural equation model examining the indirect effect of time on scores on the Liebowitz Social Anxiety Scale (LSAS), via a process variable. Filled circles indicate paths specified as random. Raised arrows indicate residuals.

All variables were measured at the lower level as per the procedure described by Bauer et al. (2006). Lagged scores were used to incorporate the temporal precedence of the process variable (mediator), where the LSAS scores at any given assessment timepoint (time j) were regressed on the process variable scores at the previous assessment point (time j-1; see Fig. 1). LSAS scores from the first week of therapy were therefore not included due to the absence of prior scores on the process variables. LSAS data from all other available assessment points were included given the model could incorporate the time gaps between sessions.

Models were computed using MPlus version 7.0 (Muthén and Muthén, 1998–2015) and R version 3.4.3 (R Core Team, 2023) using the R package ‘MplusAutomation’ (Hallquist and Wiley, 2018). Models used robust maximum likelihood estimation (MLR). Paths *a* and *c’* (See Fig. 1) were specified as random to allow variation across participants, while path *b* was modelled as fixed given that the influence of a process variable on the dependent variable must be consistent across participants (Mörtberg et al., 2015). To avoid the conflation of within- and between-subjects variance, group mean centering of the independent and process variables was used to disaggregate them into their within- and between-level components. The participant (group) mean-centered scores, and the participant mean scores across all timepoints represented the within and between components of these variables, respectively, and were entered separately into the model (see Hoffart et al., 2016; Preacher et al., 2010). The intraclass correlation coefficients for each model indicated there was sufficient between-subject variance to justify multilevel analysis (ICC = 0.34–0.58). Both *p*-values and confidence intervals of parameter estimates were reviewed to assess statistical significance.

Percent mediation (P_M_) was used to estimate the strength of any mediated effects as described in Kenny et al. (2003) and Moscovitch et al. (2005); P_M_ = 100 × [((*ab* *+* *c’* *+* *σab)-c’*)/(*ab* *+* *c’* *+* *σab*)], where *a*, *b*, and *c’* represent the path coefficients, and *σab* is the covariance between *a* and *b*. As path *b* was specified as fixed, and the covariance between a random and fixed path equals zero, the formula simplifies to P_M_ = 100 × (*ab/ab* *+* *c’*), and the indirect effect to *a × b* (see Mörtberg et al., 2015).

The direction of mediated effects was further examined using a series of models that reversed the process and outcome variables. These models used LSAS at time j-1 as the potential mediator, and self-focused attention, negative social cognitions, or depressed mood at time j as the dependent variable. Models were otherwise as shown in Fig. 1.

## 3 Results

Overall findings from the trial are reported in Clark et al. (2023). For the purposes of the present study, it is noted that LSAS, SCQ, self-focused attention and PHQ scores all showed significant decreases over the course of therapy in both the CT-SAD and iCT-SAD treatment groups (see Clark et al., 2023 Tables 3 and S4).

### 3.1 Model results

In both CT-SAD and iCT-SAD negative social cognitions and self-focused attention showed significant indirect effect estimates, indicating these variables mediated the effect of time on social anxiety as measured by the LSAS (see Table 1 for path coefficients and indirect effect estimates). The significant and negative path *a* coefficients highlighted that as time in therapy increased, scores on the process variables decreased, with the significant, positive path *b* coefficients indicating that these lower scores predicted lower social anxiety at the following assessment.

**Table 1:** Unstandardised Path Coefficients, Indirect Effect Estimates, and Random Slope Variances for the Three Process Variables Assessed, Across CT-SAD and iCT-SAD.

	CT-SAD				iCT-SAD			
	Estimate	SE	*p*	P_M_	Estimate	SE	*p*	P_M_
	Self-focused attention							
a	−0.014	0.001	<0.001		−0.012	0.001	<0.001	
b	4.800	0.743	<0.001		3.584	0.557	<0.001	
c’	−0.213	0.018	<0.001		−0.185	0.018	<0.001	
Var_a_	<0.001	<0.001	<0.001		<0.001	<0.001	<0.001	
Var_c’_	0.008	0.002	<0.001		0.014	0.005	0.008	
Indirect effect ab	−0.068	0.013	<0.001	24	−0.042	0.009	<0.001	19
	Negative social cognitions							
a	−0.017	0.001	<0.001		−0.015	0.001	<0.001	
b	8.089	0.633	<0.001		6.621	0.708	<0.001	
c’	−0.143	0.016	<0.001		−0.129	0.015	<0.001	
Var_a_	<0.001	<0.001	<0.001		<0.001	<0.001	<0.001	
Var_c’_	0.006	0.002	0.003		0.010	0.004	0.006	
Indirect effect ab	−0.138	0.014	<0.001	49	−0.098	0.014	<0.001	43
	Depressed mood							
a	−0.021	0.003	<0.001		−0.021	0.003	<0.001	
b	1.533	0.374	<0.001		0.626	0.280	0.025	
c’	−0.249	0.018	<0.001		−0.214	0.018	<0.001	
Var_a_	<0.001	<0.001	<0.001		<0.001	<0.001	0.004	
Var_c’_	0.010	0.003	<0.001		0.016	0.005	0.004	
Indirect effect ab	−0.032	0.009	<0.001	11	−0.013	0.007	0.055	ns

*Note.* Path a represents the effect of time on the mediator variable. Path b represents the effect of the mediator on LSAS score at the subsequent assessment (with time held constant). Path c’ represents the effect of time on LSAS score controlling for the effect of the mediator. CT-SAD = cognitive therapy (face-to-face), iCT-SAD = internet-based cognitive therapy, SE = standard error, P_M_ = percent mediation (i.e. the percentage of the total effect of time on LSAS score that is accounted for by the mediated path ab), Var = variance.

Depressed mood showed significant mediation in CT-SAD but not iCT-SAD. Inspection of the percent mediation values indicated that negative social cognitions showed the strongest mediation effect in both treatment formats, and that the mediating effect of depressed mood in CT-SAD was weaker than that of the other two process variables examined. The percent mediation estimates for iCT-SAD were marginally smaller than those of the equivalent models in the CT-SAD group.

### 3.2 Models reversing mediator and outcome

In both CT-SAD and iCT-SAD, the reversed models, which swapped the mediator and outcome variables but maintained the time-lag component, were significant for self-focused attention and negative social cognitions (see Table 2). This indicated that lower social anxiety scores were strongly associated with lower self-focused attention and negative social cognitions at the following assessment. The model for depressed mood was significant in CT-SAD but not iCT-SAD, indicating that lower social anxiety scores were associated with improved mood at the following assessment, but only in the face-to-face treatment format. In general, the percent mediation values indicated strong mediated effects in this direction.

**Table 2:** Unstandardised Path Coefficients, Indirect Effect Estimates, and Random Slope Variances for the Models Reversing the Temporal Order of Mediator and Outcome.

	CT-SAD				iCT-SAD			
	Estimate	SE	*p*	P_M_	Estimate	SE	*p*	P_M_
	Self-focused attention							
a	−0.301	0.018	<0.001		−0.243	0.020	<0.001	
b	0.025	0.004	<0.001		0.028	0.003	<0.001	
c’	−0.005	0.001	<0.001		−0.003	0.001	0.003	
Var_a_	0.013	0.003	<0.001		0.017	0.007	0.010	
Var_c’_	<0.001	<0.001	<0.001		<0.001	<0.001	<0.001	
Indirect effect ab	−0.007	0.001	<0.001	60	−0.007	0.001	<0.001	67
	Negative social cognitions							
a	−0.302	0.018	<0.001		−0.244	0.020	<0.001	
b	0.035	0.003	<0.001		0.032	0.003	<0.001	
c’	−0.004	0.001	<0.001		−0.006	0.001	<0.001	
Var_a_	0.013	0.003	<0.001		0.017	0.006	0.009	
Var_c’_	<0.001	<0.001	<0.001		<0.001	<0.001	<0.001	
Indirect effect ab	−0.010	0.001	<0.001	71	−0.008	0.001	<0.001	57
	Depressed mood							
a	−0.302	0.018	<0.001		−0.245	0.020	<0.001	
b	0.044	0.010	<0.001		0.016	0.011	0.134	
c’	−0.003	0.003	0.326		−0.015	0.003	<0.001	
Var_a_	0.013	0.003	<0.001		0.017	0.007	0.011	
Var_c’_	<0.001	<0.001	0.004		<0.001	<0.001	0.001	
Indirect effect ab	−0.013	0.003	<0.001	81	−0.004	0.003	0.159	ns

*Note.* Path a represents the effect of time on the mediator (i.e. LSAS at time j-1). Path b represents the effect of this mediator on self-focused attention, negative social cognition, or depressed mood scores at the subsequent assessment. Path c’ represents the effect of time on the outcome variable controlling for the effect of the mediator. CT-SAD = cognitive therapy (face-to-face), iCT-SAD = internet-based cognitive therapy, SE = standard error, PM = percent mediation (i.e. the percentage of the total effect of time on LSAS score that is accounted for by the mediated path ab), Var = variance.

### 3.3 Exploring treatment type as a moderator

**Fig. 2:**
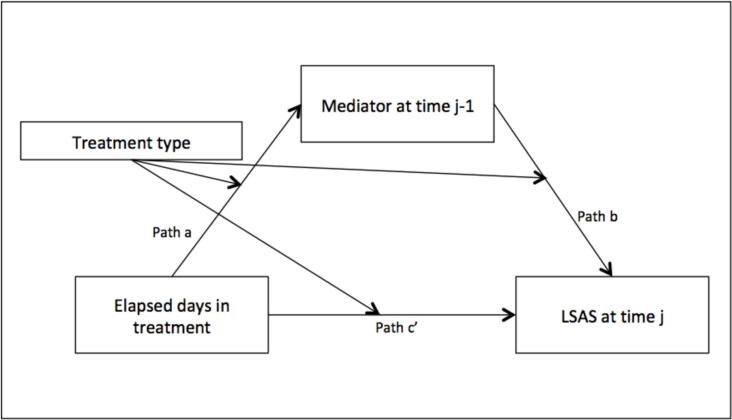
Moderated mediation model examining the influence of treatment type on the three paths shown. LSAS = Liebowitz Social Anxiety Scale – Self-report.

Although the results from the above models were generally similar between the two treatment types, an exploratory test of the influence of treatment type on the mediation relationship (moderated mediation) was performed to examine this directly. Initially, multilevel moderated mediation models (examining the effect of the moderator on the *b* path) both with and without disaggregation of the within- and between-subject variances were attempted. However, the complexity of these models led to convergence problems given the sample size. Therefore a series of single-level models were run, assessing moderated mediation as shown in Fig. 2, examining the effect of the moderator on all paths (Model 59 using the PROCESS tool, see Hayes, 2013).

Results of these models (see Table 3) were consistent with earlier findings in showing evidence for significant indirect paths (mediation) for self-focused attention and negative social cognitions, in both treatment groups, and for depressed mood in CT-SAD. In this model, the mediating effect of depressed mood was also significant in iCT-SAD. The index of moderated mediation, which tests whether the difference in mediated effects between the two values of the moderator differs from zero, showed that none of the three process variables assessed showed evidence of moderated mediation. This indicated that the mediation relationship did not significantly differ between the two treatments.

**Table 3:** Unstandardised Path Coefficients, Parameter Estimates, and Indices of Moderated Mediation for the Process Variables Assessed.

	Self-focused attention					Negative social cognitions					Depressed mood				
	Estimate	SE	*p*	Lower CI	Upper CI	Estimate	SE	*p*	Lower CI	Upper CI	Estimate	SE	*p*	Lower CI	Upper CI
a	−0.012	0.001	<0.001	−0.014	−0.010	−0.016	0.001	<0.001	−0.017	−0.014	−0.019	0.002	<0.001	−0.024	−0.015
b	7.822	0.479	<0.001	6.883	8.761	10.151	0.429	<0.001	9.310	10.992	2.258	0.223	<0.001	1.821	2.695
c’	−0.154	0.013	<0.001	−0.179	−0.130	−0.095	0.012	<0.001	−0.119	−0.072	−0.205	0.013	<0.001	−0.231	−0.180
Int_1	0.003	0.001	0.032	<0.001	0.005	0.004	0.001	0.004	0.001	0.006	0.004	0.003	0.289	−0.003	0.010
Int_2	−0.406	0.652	0.534	−1.685	0.874	0.008	0.621	0.990	−1.211	1.227	−0.446	0.282	0.113	−0.998	0.106
Int_3	0.034	0.018	0.054	−0.001	0.069	0.029	0.017	0.079	−0.003	0.062	0.045	0.018	0.015	0.009	0.081
R^2^_Mediator_	0.161					0.274					0.132				
R^2^_Dependent_	0.473					0.586					0.368				
Direct_CT_	−0.154	0.013	<0.001	−0.179	−0.130	−0.095	0.012	<0.001	−0.119	−0.072	−0.205	0.013	<0.001	−0.231	−0.180
Direct_iCT_	−0.120	0.012	<0.001	−0.144	−0.096	−0.066	0.012	<0.001	−0.089	−0.043	−0.161	0.013	<0.001	−0.186	−0.135
Indirect_CT_	−0.095	0.009	†	−0.113	−0.078	−0.158	0.010	†	−0.178	−0.138	−0.044	0.007	†	−0.057	−0.031
Indirect_iCT_	−0.069	0.010	†	−0.090	−0.049	−0.123	0.017	†	−0.155	−0.090	−0.028	0.006	†	−0.041	−0.017
Mod-Med	0.026	0.014	‡	−0.002	0.052	0.035	0.020	‡	−0.004	0.072	0.015	0.009	‡	−0.003	0.033

*Note.* a = the effect of time on the mediator; b = the effect of the mediator on LSAS score (including time lag); c’ = the effect of time on LSAS score controlling for the effect of the mediator; Int_1 = the moderation effect of treatment type on the a path; Int_2 = the moderation effect of treatment type on the b path; Int_3 = the moderation effect of treatment type on the c’ path; Direct_CT_ = estimate of the c’ path for the CT-SAD group; Direct_iCT_ = estimate of the c’ path for the iCT-SAD group; Indirect_CT_ = estimate of the indirect (ab) effect for the CT-SAD group; Indirect_iCT_ = estimate of the indirect (ab) effect for the iCT-SAD group; Mod-Med = index of moderated mediation; CI = Confidence Interval (95 %). For Indirect effects and the index of moderated mediation, SE and CI estimates were obtained through bias-corrected bootstrapping.

†. Significant upon inspection of confidence interval

‡. Nonsignificant upon inspection of confidence interval.

The moderator showed a significant effect on the *c’* path in the depressed mood model, where the CT-SAD group showed a slightly stronger relationship between time and LSAS compared to iCT-SAD. In the self-focused attention and negative social cognitions models, the moderator had a significant influence on the *a* path indicating a stronger negative relationship between time in treatment and scores on the mediator in CT-SAD compared to iCT-SAD. This suggests that self-focused attention and negative social cognitions may have decreased more rapidly in CT-SAD. The moderator did not show a significant effect on any other individual paths.

## 4 Discussion

In iCT-SAD, self-focused attention, and negative social cognitions were found to be mediators of the relationship between time in therapy and social anxiety, such that scores on these process variables predicted the level of social anxiety at the subsequent assessment point. As the same process variables showed significant effects of similar strength in face-to-face CT-SAD, the results suggest that the CT-SAD and iCT-SAD protocols assessed here appear to have their clinical effects through similar mechanisms. The moderated mediation results supported this conclusion, indicating that the mediation relationships did not significantly differ between the two treatment types, across any of the process variables assessed. This provides initial evidence to suggest it is the therapeutic content that is of primary importance in determining clinical outcomes, rather than the format of delivering the therapy. However, as this is the first study to compare change processes between online and face-to-face formats of the same therapy protocol, further similar studies are required to draw firmer conclusions.

The present findings regarding self-focused attention are consistent with existing literature, in that studies examining this have shown support for this process variable as a mediator of outcomes (Hedman et al., 2013; Mörtberg et al., 2015; Thew et al., 2020). The findings also add to the body of work supporting the role of reduced negative social cognitions in improving social anxiety symptoms (Boden et al., 2012; Calamaras et al., 2015; Goldin et al., 2014; Gregory et al., 2018; Hoffart et al., 2012; Thew et al., 2020). Few studies of change processes have used the SCQ to assess negative social cognitions, and it is noted that one did not find evidence of mediation (Mörtberg et al., 2015). It is possible that the smaller sample size (*n* = 28), and lower overall extent of change on the LSAS (mean 34.8 points) in that study compared to the present findings may have masked a possible mediating effect. Examination of two larger (>80) samples from routine clinical practice found support for SCQ scores as a mediator of outcomes (Thew et al., 2020).

Examined as a ‘control’ variable, depressed mood was not supported as a mediator in iCT-SAD, but was significant although comparatively weak in CT-SAD. The moderated mediation model suggested mediation was present in both treatments, with no difference between them, though the indirect effect estimates also suggested a weaker effect compared to the other variables examined. This is consistent with previous studies examining depression as a mediator, which found only weak effects (Moscovitch et al., 2005; Shalom et al., 2024). The findings therefore offer some support for the specificity of the two theoretically derived mediators (self-focused attention and negative social cognitions), and consequently for the cognitive model underpinning the therapy (Clark and Wells, 1995). However, work to further explore the role of depression in mediating clinical outcomes would be valuable, particularly as comorbid depression symptom levels are likely to show variability among people with SAD, and the degree to which these symptoms are addressed directly in treatment may also vary. If further evidence of mediation is established, studies manipulating the extent or timing of depression-focused techniques during treatment would help to produce prospective experimental evidence of whether the mediator has an influence on outcomes. It is also plausible that the findings in relation to depression may be being driven by specific cognitive processes such as rumination, so further work to examine such processes would be valuable. The use of a control mediator to examine specificity and support causal inference (see Preacher, 2015) represents a strength of the current study, though is uncommon within the psychotherapy mediation literature and could be considered further in future studies.

The finding that the ‘reverse’ mediation models were also significant in both treatment groups, with high percent mediation values, indicated that the level of social anxiety strongly predicted subsequent self-focused attention and negative social cognitions in both treatment groups, and depressed mood in the CT-SAD group. As self-focus and negative cognitions are key features of the clinical presentation of SAD, it is perhaps not surprising these continue to reduce following improvements in social anxiety. Taken together, the findings suggest a cyclical pattern of improvement, where changes in either the process variables or social anxiety lead to subsequent changes in the other. The fact that the therapy protocols contain interventions that target self-focused attention and negative cognitions directly suggests it is most likely that initial change in these variables precedes change in outcome, but further investigation of how such processes change across different phases of treatment would be helpful to understand this in finer detail. It is possible that the difference between CT-SAD and iCT-SAD in the reverse models for depressed mood may reflect the influence of some participants in the CT-SAD group with high depression scores. Future studies of larger cohorts could usefully explore whether comorbidity has a moderating influence on the mediation effects observed.

From a clinical perspective the findings suggest that prioritising therapy techniques designed to target self-focused attention and negative social cognitions is likely to enhance clinical outcomes and that this is equally applicable across the two treatment formats. These techniques include attention training to promote external rather than self-focus in social situations, and the use of cognitive strategies and behavioural experiments to examine, test, and reframe negative social cognitions.

### 4.1 Limitations

The present findings may be limited by the fact that self-focused attention was measured using a two-item mean. Further work to develop more sophisticated measures of this construct would be beneficial for future studies. Secondly, it remains possible that other factors may be mediating the effect of time on social anxiety in the present therapies but were not assessed. Future studies could usefully explore avoidance, safety behaviours, anticipatory worry, and post-event rumination as potential mediators using psychometrically sound measures. There is also the possibility of an unknown third variable influencing both the mediators and outcome as a function of time, meaning that the mediators and outcome may not be directly associated. However, these limitations are common to mediation designs and are not easily avoided. Given the present sample size it was not possible to run a more complex model containing the three mediators together, meaning the findings are not fully able to demonstrate their relative influence beyond the percent mediation statistics given. These values suggested that negative social cognitions were the strongest mediator, and this result was supported by the moderated mediation models, where the model containing negative social cognitions explained the largest amount of variance in LSAS scores (R^2^). Further work in larger datasets could usefully examine models incorporating these process variables in parallel. It is also possible that the process variables may influence each other in producing their effects on social anxiety, and this could not be examined in the present sample. Lastly, as outlined above, as the moderated mediation models were run using a single-level structure, they were not able to distinguish within- and between-subject variance. It is noted however that the findings were consistent with the multilevel models examined for each treatment condition separately.

### 4.2 Conclusion

Overall, this work has demonstrated that an online treatment format showed similar processes of change to the equivalent treatment delivered face-to-face. Self-focused attention and negative social cognitions mediated subsequent reductions in social anxiety. This process appeared to be reciprocal, leading to a ‘virtuous circle’ where participants experience less social anxiety, fewer and less powerful negative social cognitions, and show a greater external focus of attention as they progress through therapy. The findings suggest that efforts to improve the efficacy and efficiency of SAD interventions by targeting these process variables should therefore be equally applicable to online and face-to-face interventions.

## Declaration of competing interest

The authors declare that they have no known competing financial interests or personal relationships that could have appeared to influence the work reported in this paper.
